# A systematic review and narrative synthesis of the experiences with caring for older people living with dementia in Sub-Saharan Africa

**DOI:** 10.1186/s12877-022-03668-2

**Published:** 2022-12-13

**Authors:** Purity Mwendwa, Brian Lawlor, Thilo Kroll, Aoife De Brún

**Affiliations:** 1 UCD Centre for Interdisciplinary Research, Education, and Innovation in Health Systems (UCD IRIS), School of Nursing, Midwifery & Health Systems, University College Dublin, Dublin, Ireland; 2 Global Brain Health Institute, Trinity College Dublin, Dublin, Ireland

**Keywords:** Dementia, Older people, Family caregivers, Informal carers, Systematic review, Sub-saharan Africa

## Abstract

**Background:**

In low- and middle-income countries, including sub-Saharan Africa little is known about the experiences with caring for people living with dementia. The purpose of this systematic review and narrative synthesis was to examine the experiences with caring for older people living with dementia at home.

**Results:**

In total, 366 abstracts were identified and following screening, 19 studies were included in the synthesis. Six themes were identified: conceptualising dementia, caregiving arrangements, the impact of caregiving, caregiver identity and role, managing caregiving, unmet caregiver needs.

**Conclusion:**

There is a dearth of research in relation to caregiving for older people living with dementia in sub-Saharan Africa. There is need for better information campaigns and support programs directed at family and professional caregivers in this context.

**Supplementary Information:**

The online version contains supplementary material available at 10.1186/s12877-022-03668-2.

## Background

Dementia is an umbrella term used to describe a syndrome that is due to many different diseases that affect the brain, causing problems with memory, language and cognitive abilities that interfere with day to day function [1]. Age is the most common risk factor for developing dementia [2] although it is not an inevitable part of ageing as those at younger ages can also develop dementia [3]. Dementia is now considered a global public health concern and currently affects approximately 50 million people worldwide [4]. Alzheimer’s disease (AD) is considered the most common cause of dementia, accounting for between 60 and 80% of all cases [1]. While AD and other forms of dementia are less common in sub-Saharan Africa (SSA), they still account for a significant disease burden in older people in this region. In 2010, for example, AD was cited as a major cause of disability and mortality in people aged 70 years and above [5]. However, limited data from the region make reliable estimates of prevalence, morbidity, mortality and caregiver burden related to dementia difficult [6]. Dementia places a significant burden on individuals, caregivers and their families, particularly in low- and middle-income countries (LMICs) including SSA [7]. The majority of people living with dementia (PLWD) reside in the home, but the numbers differ hugely between LMIC and high-income countries (HICs). In LMICs, nearly 95% of PLWD reside at home compared to 60–70% in HICs. LMICs account for nearly 60% of the global informal care hours with women in these settings contributing to the highest proportion of this informal care [8]. There is a call for countries in SSA to prioritise, develop and implement policies that strengthen research and dementia care and supports [7].

SSA generally faces challenges in relation to the care of older people. Most SSA countries have to contend with fragile and under-resourced health care systems with limited health care capacities [9]. Moreover, most investment in health care systems in SSA has focused on managing infectious and childhood diseases, rather than on chronic conditions, which are more common in the older population [10]. While countries in the region have gradually begun to develop national policies aimed at addressing the health and well-being of older people [11–13], these efforts have thus far been of limited success due to poor implementation, monitoring and evaluation [14]. The African Union’s Africa Health Strategy 2016–2030 seeks to enhance regional and global health commitments in the region generally but fails to mention old-age related health conditions [15]. In SSA, most caregivers are women [16]. They are frequently the only source of support and care for PLWD in the absence of adequately resourced, integrated health and social care systems.

There is considerable evidence to show that caring for PLWD often demands substantial effort, time, and resources and hence places a significant burden on caregivers and families [17, 18]. However, much of this evidence comes from HICs [18]. The current review is grounded in a robust and transparent methodological approach to add to the limited knowledge and evidence in relation to caring for older PLWD in the SSA context. While countries in SSA are not homogenous in any respect, they share a range of challenges not the least of which is a lack of priority in managing the care of older people [10].

### Aim

The aim of this review was to address the following question: What are the experiences with caring for older people living with dementia in sub-Saharan Africa? In this review we focused on informal caregivers and we used the definition of ‘caregiver’ provided by the Family Caregiver Alliance [19]. They defined a caregiver as ‘an unpaid individual (a spouse, partner, family member, friend, or neighbour) involved in assisting others with activities of daily living and/or medical tasks. We were interested in exploring 1) characteristics (study designs) of articles published on the topic, 2) characteristics of caregivers participating in the studies and 3) the experiences of being a caregiver for older PLWD in SSA.

## Method

### Protocol and registration

This systematic review with narrative thematic synthesis followed the Preferred Reporting Items for Systematic Reviews and Meta-Analysis (PRISMA)-statement [20]. Inclusion and exclusion criteria were developed by the research team (Table 1). The review protocol was registered with PROSPERO (CRD42019132547) [21] and published in June 2019.

**Table 1:** Inclusion and exclusion criteria

Inclusion criteria	Exclusion criteria
Studies relating to the experiences of caring for an older PLWD in SSA	Studies on the experiences of the PLWD
Studies that identify caregivers as study participants (caregivers include family members and relatives of the PLWD, community members, paid and unpaid caregivers providing care for someone diagnosed with dementia in the home setting	Studies that include professionals or organisations’ experiences with a PLWD
All research methods	Publications without reported empirical data, such as commentaries, letters, and editorials
Publications in English	Publications in other languages
Publications must clearly locate the study population in the SSA context	Publications not referring to the SSA context

### Literature search

Six databases (PubMed, CINAHL, EMBASE, PsycINFO, the Cochrane Library and Web of Science) were searched from inception to May 2020 using MeSH headings and key words. Existing systematic reviews informed the construction of the search strings [6, 22]. We used the Boolean combinations of alternative terms for ‘dementia’, ‘caregiving’, ‘older people’, and ‘sub-Saharan Africa’.

Grey literature databases were also searched for relevant studies (Google Scholar, The Alzheimer’s Association Virtual Library, The World Health Organisation (WHO) and DATAD - Database of African Theses and Dissertations). In Google Scholar, the first 20 pages of the search results, sorted by relevance were screened for relevant studies [23]. We then used the terms ‘dementia’, ‘caregiving’, ‘older people’, and ‘sub-Saharan Africa’ to search the rest of the databases for relevant articles, reports, and theses. In addition, reference lists and citation searches of included studies were examined. Hand searches were undertaken in the following journals from inception to May 2020: South African Family practice, West African Journal of Medicine, and the South African Journal of Psychology. The full search strategies for all databases can be found in Table 2. The original electronic database searches were conducted in March 2019.

**Table 2:** Search strategy terms

Population	Key terms	Context
caregiver* care-giver* relative family* families* dependent* kin* spouse* parent* folk* “spouse-caregiver”*	Dementia OR Delirium “Wernicke Encephalopathy” “Cognitive Disorder*” dement* alzheimer* “lewy* bod*” OR deliri* “chronic cerebrovascular” “organic brain disease” “organic brain syndrome” “normal pressure hydrocephalus” “shunt*” “benign senescent forgetfulness” “cerebr* deteriorat*” “cerebral* insufficient*” “pick* disease” creutzfeldt jcd cjd huntington* “binswanger*” korsako	“Africa” “sub-Saharan Africa” “Western Africa” “Central Africa” “Eastern Africa” “Southern Africa “

### Eligible studies, inclusion, and exclusion criteria

We included qualitative, quantitative and mixed-methods studies that were relevant to the review question. The review included articles found in peer-reviewed journals and grey literature databases written in the English language. Table 1 provides a detailed description of the inclusion and exclusion criteria.

### Study selection

Search results from each database were exported to Rayyan (https://rayyan.qcri.org/), a web-based tool designed to help researchers working on knowledge synthesis projects in the screening and selection of relevant studies after which duplicates were removed. Two researchers (PM and ADB) independently screened half of the titles and abstracts to exclude articles that did not meet the inclusion criteria. Next, full texts were retrieved and independently assessed for eligibility by the two reviewers. Disagreements were discussed until consensus was reached. Reference lists were checked to identify additional relevant studies for the synthesis.

### Quality assessment

Two of the authors (PM and TK) independently assessed each publication for comprehensiveness of reporting. We used the Mixed Methods Appraisal Tool (MMAT) [24] which was specifically designed for appraising mixed-methods reviews. All studies, regardless of quality assessment outcome were included in the review. Table 3 provides the scores assigned to each paper based on agreed outcomes from the two authors.

**Table 3:** Characteristics of included articles

Author, Year	Country	Aim of the study	Diagnosis Identified	Type of Participants and sample size	Study design	Methodological approach	Quality Score
1) Agudu 2017 [25]	Ghana	To explore and describe the experiences of family caregivers caring for patients with dementia	Dementia	Family caregivers *n* = 10 Female *n* = 9 Male *n* = 1 Age 25–74	Qualitative	Face-to-face in-depth interviews	***** (100%)
2)Agyeman et al. 2019 [26]	Ghana	To explore the sociocultural beliefs, understandings, perceptions, and behaviours relating to living with dementia in Kintampo	Dementia	Family caregivers *n* = 10 Female *n* = 6 Male *n* = 4	Qualitative-Case studies	In-depth interviews	***** (100%)
3) Bosch 2014 [27]	South Africa	To explore the needs and experiences of caregivers of persons with Alzheimer’s disease living in black rural communities in Mpumalanga, South Africa	Alzheimer’s Disease	Caregivers *n*-11 Female *n* = 10 Male *n* = 1 Age 23–82	Qualitative	Individual interviews	***** (100%)
4) Deist and Greeff 2017 [28]	South Africa	To identify factors associated with family resilience in families caring for a parent with dementia.	Dementia	Families in which adult children were caring for a parent with dementia. *n* = 47 Female *n* = 38 Male *n* = 9 Age 20–64	Mixed methods	Quantitative survey and qualitative interviews	***** (100%)
5) Deist and Greeff [29]	South Africa	To explore factors associated with family resilience in families caring for a family member with dementia	Dementia	Families in which a spouse was caring for a partner with dementia. *n* = 44 Female *n* = 29 Male *n* = 15 Age 43–90	Mixed methods	Quantitative survey and qualitative interviews	***** (100%)
6) Dotchin et al. 2014 [30]	Tanzania	To document the burden of caregiving for people with Parkinson’s disease (PD) and dementia.	Dementia	Caregivers for PLWD Female *n* = 38	Quantitative	Questionnaire/ survey	**** 80%
7) Gurayah 2015 [31]	South Africa	To explore the phenomenon of caregiving for people with dementia in a rural context in South Africa.	Dementia	Caregivers *n* = 5 4 Females (3 daughters and 1 wife of the PLWD) 1 Son of the PLWD Age 46–68	Qualitative	Individual interviews	***** (100%)
8) Hendricks-Lalla and Pretorius 2020 [32]	South Africa	To explore the experiences of male familial caregivers of persons with Alzheimer’s disease from low socio-economic status	Alzheimer’s disease	Male family caregivers of persons with Alzheimer’s disease *n* = 11 (6 children (sons), 5 spouses (husbands) Age 48–83	Qualitative	Semi-structured interviews	***** (100%)
9) Mayston et al. 2017 [33]	Nigeria = only country of interest	To explore the social and economic effects of caring for an older dependent person, including insight into pathways to economic vulnerability.	Dementia	*n* = 24 sites and *n* = 60 interviews total across 4 countries In Nigeria: 7 household (cases) with 20 interviews *n* = 14 with caregivers *n* = 6 with older people- not	Qualitative (Case studies across urban and rural sites)	Interviews with family members of older dependent persons (some with dementia)	***** (100%)
10) McFarland 2010 [34]	Botswana	To share a personal experience of caring for a terminally ill family member who was afflicted with Pick’s Disease, a type of dementia	Pick’s Disease	1 female caregiver	Qualitative research	Self-reflection of a personal experience with Pick’s Disease	****** (100%)
11) Mkhonto and Hanssen [35]	South Africa	To explore and describe the link between culture and dementia care with the focus on the influence of the belief in dementia as witchcraft and people with dementia as witches	Dementia/Alzheimer’s disease	*n* = I8 close family members - 16 female and 2 male) *n*-19 nurses caring for patients with dementia	Qualitative	in-depth qualitative interviews	***** (100%)
12) Mahomed and Pretorius 2020 [36]	South Africa	To explore the needs of male caregivers of people with Alzheimer’s disease (AD) by ascertaining the availability and utilisation of Alzheimer’s disease-related resources in low-income communities in the Western Cape	Dementia/Alzheimer’s disease	Males familial caregivers of persons with Alzheimer’s disease *n* = 11 18 mean age = 61.18 Co-residents 5 sons; 5 husbands ;1 son-in law Age 49–87	Qualitative	Semi-structured interviews	***** (100%)
13) Mushi et al. 2014 [37]	Tanzania	To explore the social cultural beliefs surrounding dementia and the life experience of people with dementia and their caregivers.	Dementia	25 paired interviews with PWD and their caregivers and 16 interviews with caregivers alone Caregivers *n* = 16 Females = 11 Males *n* = 5 Age 19–58	Cross-sectional qualitative design	Qualitative interviews	***** (100%)
14) Potgieter et al. 2012 [38]	South Africa	To identify and describe the changes in the time perspective of persons caring for a spouse diagnosed with Alzheimer’s disease	Dementia	Female participants *n* = 40 Age 53–82 years	Mixed methods	Qualitative and quantitative instruments	***** (100%)
15) Potgieter and Heyns 2006 [39]	South Africa	To identify stressors and strengths reported by female caregivers of spouses diagnosed with Alzheimer’s disease	Dementia	Caregivers (female) *n* = 8 Age 37–71	Mixed methods	Qualitative, interviews triangulated with quantitative measures	***** (100%)
16) Pretorius et al. 2009 [40]	South Africa	To explore the experiences of men caring for spouses suffering from dementia from a salutogenic perspective	Dementia	Men caring for a spouse with dementia. *n* = 10 Age- 61–86	Mixed methods	Semi-structured interviews and quantitative questionnaire	**** (80%)
17) Prince and 10/66 Dementia Research Group 2004 [41]	24 centres in developing countries worldwide, including Nigeria	To comprehensively assess the care arrangements for people with dementia in developing countries	Dementia	706 interviews with people with dementia and their caregivers *n* = 20 in Nigeria. Female *n* = 19 Male *n* = 1 Age 40–64	Quantitative descriptive	Descriptive and comparative study	(**** 80%)
18) Smith et al. 2018 [42]	South Africa	To explore and describe the transition experiences of eight family members regarding how they became caregivers of their relatives with dementia.	Dementia	Family caregivers *n* = 8 Females *n* = 7 male *n* = 1 1 male- spouse 4 daughters;1 spouse; 1 sister; 1 daughter Age: 47–72 Mean age = 60.6	Qualitative Phenomenological design	Visual participatory methods in the form of collages and interviews	***** (100%)
19) Raphael et al. 2017) [43]	Nigeria	To explore the impact of educational intervention on the family caregivers for older people with dementia	Dementia	Family caregivers *n* = 56 Female *n* = 51 Male *n* = 5 Age 18–72	Quantitative	quasi-experimental: pre and post-test design	Not rated

### Data extraction

A data extraction form was developed to extract the main details, concepts, sub-concepts, and relevant findings from each paper. We included data relating to study characteristics (study information, study methods and type of participants) key findings that related to the review questions (caregiver characteristics (for example, spouse, adult child, other family members, peers, and neighbours) and reported findings on experiences of caregiving and/or impact of interventions (coded and organised under thematic headings). The key findings were then collated in a spreadsheet for thematic analysis.

### Analysis and narrative synthesis

To analyse the data, we adhered to the principles of thematic analysis and synthesis [44], a three-stage iterative process that entails coding, identification of themes and the generation of analytical themes. The initial stage of the analysis entailed line-by-line coding of data (quotations, results, and findings) from the selected studies that made sense of the experiences with caring for older PLWD in SSA. In keeping with the thematic synthesis approach, we identified related codes and combined these into broader descriptive themes. The themes were extracted and analysed inductively and hence were not guided by a predetermined set of codes [45]. For quantitative studies, we identified common patterns within independent variables in relation to our study’s research question and these were merged with those from qualitative studies. To describe the results, we used the narrative synthesis method, an approach that entails systematically synthesising and explaining findings from many studies using text and words [46].

## Results

### Study selection

**Fig. 1:**
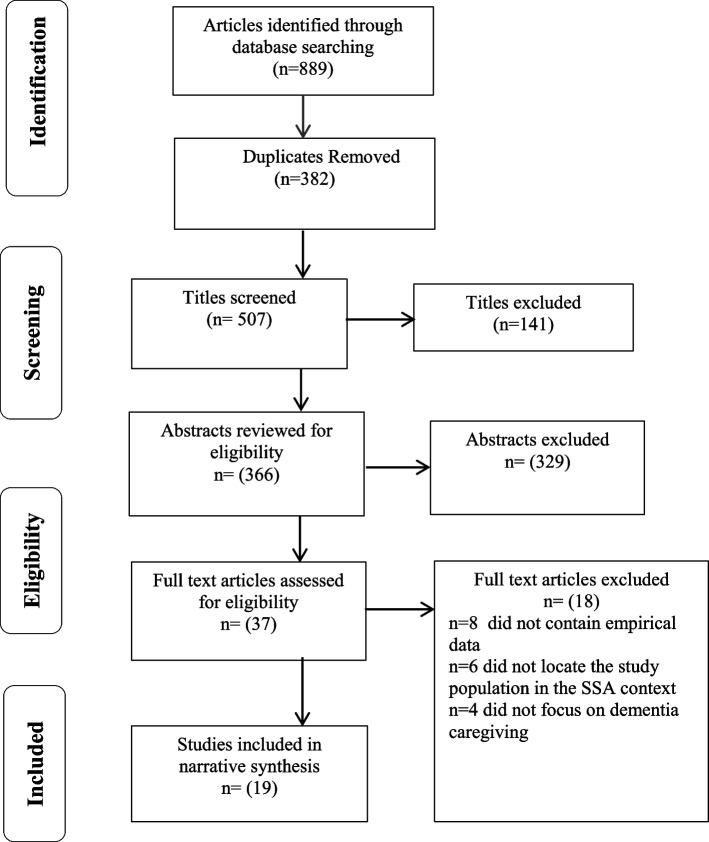
Flow diagram of the systematic review process

We identified 889 records and of these, 382 duplicates were removed. A total of 507 titles were screened for eligibility and 141 were excluded. Following abstract screening, 329 were eliminated leaving 37 records which were subjected to full text screening. A further 18 records were eliminated on the following basis: they did not contain empirical data (*n* = 8), others, (*n* = 6), did not locate the study population in the SSA context and (*n* = 4) did not focus on dementia caregiving. In total, 19 studies met the eligibility criteria and were included in the review. Figure 1 provides a summary of the screening and selection phases of the review process.

### Participant and study characteristics

The studies reported the experiences of 378 caregivers. Most caregivers (287) were female relatives (spouses, daughters, daughter in-laws, step-daughters, granddaughters, sisters, sister- in laws, maternal aunts) and 87 male caregivers (spouses, sons and brothers), one study [33] did not disaggregate the data by gender. Caregivers were aged between 19 and 90 years. Care recipient’s diagnosis was identified as dementia (13 Studies), Alzheimer’s disease (five studies), and Picks Disease (one study).

Out of the 19 studies included, 11 were qualitative in design [25–27, 31–37, 42], five were mixed-methods [28–30, 38–40, 43], three were quantitative [41, 47, 48]. Most studies (*n* = 11) were conducted in South Africa, two in Tanzania, two in Ghana, one in Nigeria and one in Botswana. Two studies [33, 41] were conducted across multiple sites including Nigeria. A full description of the studies is provided in Table 3.

### Quality appraisal

Fifteen papers met all the five quality criteria of the MMAT, and these received a five-star rating (*****= 100%). Three papers received a rating of four stars (****= 80%). One article did not meet the quality criteria and received no rating. All studies regardless of quality were included in the review (Table 3).

### Narrative synthesis

The 19 studies (participant *n* = 378) were included in the narrative synthesis. Themes extracted from the studies were synthesised into six broader descriptive themes (see Table 4).

**Table 4:** Themes arising from the thematic synthesis

Themes
1.Conceptualising dementia
2.Caregiving arrangements
3.Impact of caregiving
4.Caregiver identity and role
5.Managing caregiving
6.Unmet caregiver needs

#### Conceptualising dementia

The understanding of dementia as a medical condition and the knowledge caregivers, family members and the community had about potential causes was described as limited in a number of studies [27, 30, 31, 35, 37]. Dementia was commonly regarded as a normal part of ageing and there was no local term to describe it [26, 30, 37]. Based on this limited understanding, some caregivers described feeling devastated and in shock upon learning of the diagnosis of their loved one [32]. However some participants had received caregiver training [27] and others [36] had received  basic information about AD which they described as very helpful. However, male caregivers were described as reluctant to know details of the disease and its progression and preferred instead to take one day at a time [40]. In some studies [26, 35], the condition was attributed to witchcraft, based on the belief that the decline in cognitive functions was a result of bewitchment. Low rates of diagnosis was reported in a number of studies [26, 27, 30, 35–37].

#### Caregiving arrangements

Female relatives were predominantly the hands-on caregivers, performing daily tasks related to caring for the PLWD. Daughters, sisters, wives, granddaughters, daughter-in laws and nieces were associated with this role in a number of studies [25–29, 31, 34, 35, 37–39, 41–43]. In a smaller number of studies men were identified as the hands-on caregivers [25, 31–33, 36, 37, 40, 42]. Caregivers provided care on a daily basis and in most studies, the caregiver lived in the same home with the PLWD [25, 26, 31, 37, 41, 47–49]. Because of cultural norms associated with caring for older adults in the African setting, PLWD were not placed in residential care [34, 47], although one study included caregivers of PLWD living both at home and at a long-term care facility [42]. One study noted that no residential care facilities were available for PLWD within the study location [27]. In a few studies [28, 29, 33, 41] paid caregivers were engaged in offering supplemental help and were called upon particularly if care needs in the house increased (for example a second person in the home required care), or if the health of the PLWD deteriorated and the main caregiver could not cope [33].

#### The impact of caregiving

The caregiving role was described as stressful, demanding, difficult and hence emotionally, physically, and financially exhausting. Caregiving became increasingly difficult as the PLWD became dependent and care needs increased [27]. A number of studies reported relatively high levels of caregiver burden and strain [25, 27, 34, 37–39, 41] and hence, caregiving was associated with poor physical and psychological health outcomes [27, 30, 39]. A greater burden in females was reported in two quantitative studies [39, 40]. And in one study that explored family resilience in caring for PLWD, the age of the caregiver and that of the PLWD appeared to have a significant negative correlation with family adaptation [29]. Caregiver strain was described as lower in larger households [30], and when tasks where shared among friends and family [34] including in instances where the caregiver was resident with the PLWD [41].

The most difficult time for caregivers was before a formal diagnosis was determined, as the caregiver did not quite comprehend the changing behaviours of their family member [39]. Some found it difficult to handle the reality presented by the diagnosis and the unpredictable behavioural changes of PLWD [40, 42]. In a number of cases, caregiver strain was amplified by problematic behaviours from the PLWD including aggression, irritability and disinhibition [25, 31, 32, 47] and some participants talked of being sleep deprived [25]. The inability to communicate correctly with the PLWD proved a significant stressor for caregivers [40]. A lack of support from family members seemed to exacerbate the caregiver burden [27, 42].

Caregiving resulted in significant relationship difficulties between the caregiver and the PLWD and among family members. Being a caregiver often meant less free time [32], social isolation [27, 31] and restriction from leisure and social activities [27, 31].Those caring for a spouse described their relationship as adversely impacted and the caregiving role often created dissent around caregiving duties, which seemed to impinge on family relationships [25, 32]. Conversely, in some studies the caregiving experience strengthened relationships among family members [25]. Overall, caregivers had to adapt their personal lives and that of other family members to accommodate caring for the PLWD [42].

The burden of providing care was also exacerbated by financial difficulties. Families had to contend with additional costs relating to caring for PLWD (for example, transportation to health care facilities, the purchase of medication and other essential supplies) while fulfilling multiple family demands [25–27, 31–33]. The duration and severity of the illness as well as the family’s social-economic status intensified the economic burden [41]. As most caregivers were engaged in their role around the clock, their career development and earning opportunities were impacted [25, 38]. Additionally, caregivers, often cut down on employment or other income generating activities to focus on caregiving [26, 31, 41]. Inadequate funds meant that access to basic needs and essential services was affected [26, 32, 33]. Some caregivers incurred additional expenses to employ caregivers [28, 29, 33] and some had availed of private health care services which were more expensive than public healthcare [41]. Given the uncertainty of the progression of the illness, it was difficult to project future health care costs [26].

#### Caregiver identity and role

Some caregivers talked about how their role came about, describing it as sudden, unexpected, new and strange [42]. Becoming a caregiver was an unfamiliar role that often made them feel overwhelmed and distressed. Some described their new role as something that did not come naturally to them, while others compared their life situation as before and after the diagnosis, describing the life after diagnosis as ‘paradise lost’ [42]. For others, caregiving was generally viewed as a sense of duty and was motivated by familial expectations and the African cultural norms [27, 31]. When dementia was attributed to the normal ageing process, some found it easier to accept and get on with their caregiving responsibilities [31]. In contrast to most female caregivers, some male caregivers preferred to make sense of their caregiving experience at a cognitive rather than an emotional level [40], presumably as a way to guard against emotional burden and enhance the ability to cope with their situation.

Yet, some described their role as rewarding and a way of giving back, especially when the person being cared for was a parent or a spouse [27, 33, 40]. In one study [39], spousal caregivers talked of personal growth, describing coping well with their current situation and even excelling at tasks their spouses previously took charge of. The experience had enhanced meaningfulness in their lives and despite the enormity of their role, caregivers upheld an awareness of the potential for a future beyond their current situation [39]. The role was also viewed as a character-building experience in one study [31]. In a study with male caregivers [32], participants reported having found meaning in their role upon realising their abilities in discharging their caregiver duties. Another study described the caregiving experience as a challenging and difficult one that called for strong coping mechanisms, but one that revealed and taught the caregiver about a disease they had not heard of before [34].

#### Managing caregiving

Despite the caregiver burden articulated in most studies some caregivers were well able to cope and adapt to their new role [39, 40]. Acceptance of the diagnosis of the PLWD was deemed important to enable coping with the caregiver role [28, 29, 39]. The importance of remaining positive and optimistic in the face of a deteriorating situation, praying and reading the bible were regarded as key coping strategies [28, 29]. Importantly, spirituality and religion were deemed a motivator to continuing caring for the PLWD [27] and religious beliefs played a part in ensuring the caregiver stayed positive and engaged in their role [39]. However, caregivers citing religion as essential to coping were often affiliated with the same religious group and their experiences may not mirror those from other religious groups. In two mixed methods studies [28, 29], qualitative findings found that the use of support from religious and spiritual groups was important in providing strength during difficult times. However quantitative findings from these studies showed no significant correlation between the use of this support and a family’s ability to cope.

The family unit was similarly crucial in supporting the PLWD and offering support to the main caregiver(s). Most caregivers relied on the physical, material, and emotional support of family members. Male caregivers turned to their daughters or hired help to manage the practical aspects of caregiving [40]. Ideally, a larger family often meant that caregiving duties could be shared among family members [28, 29]. However, the ability for caregivers to tap into the family resource was contingent on familial bonds [28, 29] and the acceptance and understanding of the illness of their relative [39]. Quantitative findings highlighted the importance of positive communication patterns, flexibility within the family and consistent family routines [29] as key to enhance family adaptation. Financial stability and ability to manage the symptoms of the PLWD had a positive impact on the family’s ability to adapt to the new role [28].

In addition to family support, was the external social support, and professional services available to some caregivers. Support came mainly from caregiver support groups and other caregivers, friends, neighbours, paid caregivers, community and religious groups as well as health care professionals [25, 28, 32, 34]. While not all caregivers had access to a support group [27] these provided an avenue to share the caregiving experience and learn coping skills while enabling caregivers to expand their social network [29, 39, 40, 47]. In one study with male caregivers [40] few attended support groups citing the high number of female caregivers and the emotional nature of the group as inhibiting factors. In other studies, caregiving demands precluded the ability to attend support groups regularly [27, 32]. Overall caregivers who attended support groups spoke of the benefits they derived from these groups [27, 36].

The need for temporary relief from caregiving duties was articulated in a number of studies where immediate family members, friends, neighbours and paid caregivers provided respite [27, 28, 31, 34, 36]. Some families had availed of community respite-care services [27, 34, 40]. In addition, caregivers’ involvement in activities not related to caregiving served as a form of respite and contributed to enhancing the meaning and purpose in their lives [40]. Some caregivers participated in social activities in the church which served as a respite from their duties [29]. Given that most family members lacked the experience and insights into what it meant to care for a PLWD, it precluded their willingness to offer respite care [39].

Community-based supports came from several sources, including the government, religious and local community groups. In a few studies, caregivers talked of availing of government and non-governmental supported services, such as day-care and home-based care services [32, 34]. Support from religious groups came from church members and prayer groups [25, 29, 36, 37]. In the local community neighbours and, in a few cases, retirement homes, served as an important support structure for caregivers and their families [25, 27, 29]. Quantitative findings found community support to be positively correlated with a family’s coping ability [28].

A number of caregivers sought diagnosis, treatment and advice for the PLWD from modern health care services [25, 26, 36, 39, 40]. Because in some cases dementia was linked to supernatural causes, some sought traditional healers [27] and others [26, 27] used a combination of modern and traditional services, including spiritual interventions [25, 37]. In a number of cases, however, the use of modern healthcare was for ailments unrelated to dementia symptoms [37]. Most caregivers talked of the value of modern healthcare services, particularly the medications prescribed [40] and the psychotherapy offered to caregivers and PLWD, although these services were deemed expensive [39]. In other studies caregivers talked of negative perceptions with services, stigma and neglect which precluded their willingness to use such services [36].

#### Unmet caregiver needs

The burden of caregiving was heightened by the lack of knowledge and understanding of dementia, no government support [37] and a lack of accessible services or professional guidance as the illness progressed [36].In an effort to fill the knowledge gap some caregivers turned to books and the internet for information [28, 29]. The importance of psychoeducation and development of relevant caregiving skills, particularly during the initial stages of the disease, was echoed in a number of studies [31, 35–37]. The need to improve the care and support of PLWD and their families was a common theme articulated in all studies. Government support was noted as largely absent [29, 33, 36, 37, 41]. The need for government and non-governmental agencies to play a central role in providing food to families, financial support, treatment and setting up support and information centres [31, 37] were regarded as key to reduce the caregiving burden.

## Discussion

This systematic review and narrative synthesis sought to explore the experiences with caring for older PLWD in the SSA region. This review identified a relatively small number of studies (*n* = 19), the majority (*n* = 11) of which were conducted in South Africa. Only five countries in the region (South Africa, Nigeria, Ghana, Tanzania, and Botswana) are represented in the identified studies. The general dearth of research related to dementia in SSA has recently been highlighted [48]. Most studies in the current review (15 out of 19) were judged to be of high quality, although one study was assigned no score as it failed to meet the quality criteria. For the group of countries included in our study, the issues identified related with caregiving in the context of dementia are similar.

This review identified six themes related to the experiences with caring for older PLWD in SSA: conceptualising dementia, caregiving arrangements, the impact of caregiving, caregiver identity and role, managing caregiving, unmet caregiver needs. Beliefs that dementia symptoms are simply a sign of old age and not the result of a medical condition emerged in a number of studies [27, 30, 31, 35, 37]. This might explain the lack of diagnosis or low rates of diagnosis among older people presenting with cognitive decline [26, 27, 30, 35–37]. In a few studies of the current review [26, 35] the older person’s condition and cognitive decline were attributed to witchcraft, which underscores the limited understanding of dementia and is consistent with findings in other studies [6, 49]. Other studies conducted in the SSA region [6, 50] suggest that while dementia is not recognised as a medical condition in a number of settings, it presents a huge caregiver burden.

Closely linked to the knowledge gap is the stigma and discrimination associated with having dementia. This often results in social isolation [17, 51], fear of the PLWD and their family, and in some cases violence and mistreatment of older people displaying mental illness [49]. From our review, few studies [27] identified stigma as a significant problem although some caregivers talked of neglect and maltreatment of the PLWD at health care facilities [36]. There is evidence to suggest that poor understanding of Alzheimer’s disease and other dementias leads to stigma and abuse [52]. The perceived lack of stigma from the studies reviewed is likely due to the belief that dementia is a normal part of ageing and that nothing could be done about it to reverse its course [37]. While most caregivers yearned for more information and knowledge about dementia, male caregivers in one study [40] were reluctant to learn more about the disease, preferring to take the situation one day at a time. This was presumably a way of mitigating the stress and worry about the future. Other research [53] has found that male caregivers, in particular, tend to underutilise support, for example opportunities for skills development, that could improve their caregiving experience.

Consistent with the wider literature on the experiences with caring for PLWD [54, 55] caregiving was considered a family responsibility and the majority of caregivers were female relatives (spouses and daughters) who provided care on a full-time basis. This is consistent with many African traditional norms, where the expectation is for family to support and care for older and frail relatives. In some cases caregiving was a collective effort among close family relatives with duties shared within the family [25, 26, 42]. Studies conducted internationally among ethnic minorities, in which caregiving took on a cultural and collective approach [56, 57], support our review findings.

The negative physical, social, emotional and financial burden associated with caregiving has long been acknowledged in the dementia care literature [17, 18]. In our review, these negative aspects seemed to be inter-related; the complex care needs of the PLWD and the fact that providing care became increasingly difficult as the PLWD became dependent proved physically demanding [27]. Emotionally, caregivers talked of stress, worry and expressed uncertainty about the future as they struggled to understand and accept the condition of their family member [42]. Against this backdrop, the caregiving role left caregivers with little to no time for themselves or social activities, leading to social isolation and loneliness. The greatest contributor to the caregiver burden in our review was financial stress. This was mainly related to direct and health care costs, including the purchase of herbal remedies [26] straining the ability to provide basic needs for the PLWD and the family. There is evidence to show that caregivers from low income settings tend to avail of the more expensive private health care services based on their experience with poor negative services in the public sector [41].The financial situation of families was exacerbated if caregivers left employment or income generating activities to become fulltime caregivers [25].

The lack of government involvement in the care and support of the PLWD and their families was a consistent theme across reviewed studies. This finding supports others conducted in LMICs [6]. The Global Action Plan on the Public Health Response to Dementia 2017–2025 [58] acknowledges the challenges that governments face in responding to the growing number of those living with dementia globally. The approach recommends that countries adopt a public health approach to address the needs of PLWD their caregivers and families. This would require the development and implementation of national policies that specifically address dementia and dementia care that are currently either at the nascent stages of development or totally absent in most of SSA.

The importance of education and awareness raising programs to address the prevailing misconceptions around causality of dementia within families was articulated in the studies reviewed. We argue that such programs need not only target caregivers and their families, but the general public. Other studies have found locally led initiatives, in particular those delivered through religious organisations (for example, in churches and mosques) as effective in addressing sensitive and potentially stigmatised topics [59]. Such interventions need to be informed and co-designed with local community members to ensure cultural relevance and acceptability. Indeed, studies conducted in more developed contexts such as the US and Europe show the need to involve communities in developing interventions for informal caregivers in the dementia context [18].

### Strengths and limitations

While a variety of reports exist in relation to dementia in SSA, this review is the first that employed a transparent and robust, systematic evidence review and synthesis with a focus on caregiving. The current review presented here employed a robust and transparent methodological approach in assessing and synthesising the evidence-base, specifically in relation to the experiences with caring for older PLWD in SSA. The literature search involved multiple databases and sophisticated search algorithms. All publications were thoroughly screened and appraised. Yet, there is a possibility that some publications may have been missed. The review was limited to journal publications or grey literature in the English language alone. French, Portuguese, or Afrikaans language publications were not included if they did not contain an abstract written in English. Moreover, some country-specific reports in the grey literature may have been missed. The quality appraisal tool for mixed-methods publications MMAT is still undergoing further development but thus far, it is the most widely used single quality appraisal instrument to characterise the quality of quantitative, qualitative and mixed-methods studies and it has been used in similar studies [60]. Despite some of these limitations, the review provides a solid map of the current literature on caregiving for PLWD in SSA and these findings can inform the design of support interventions as well as public health messaging and advocacy on dementia in various SSA countries, as well as direct avenues of future research through demonstrating gaps in the evidence.

## Conclusion

This systematic review has highlighted the dearth of research in relation to caregiving for PLWD in SSA. Only five countries contribute experiential research to the body of evidence which limits the generalisability to all SSA. The review underscores the need for better information campaigns and support programs directed at family and professional caregivers in this context. Our review focused on the experiences of informal caregivers in the home setting. While the views and experiences of informal caregivers may differ from those working in formal settings, our findings can offer valuable evidence that would inform the development of national dementia and care policies and strategies that are currently underdeveloped in SSA. Our review underscores the importance of including the voices of the informal caregivers and the requirement that dementia plans and policy must address caregiver needs. Further, our review suggests that men are an underrepresented cohort in caregiving which may not reflect the reality on the ground. There is a need for more research with male caregivers to address these gaps.

## Supplementary Information

**Additional file 1.**

## Data Availability

The data for this review is provided under supplementary information.

## Abbreviations

AD: Alzheimer’s disease
HIC: High-income countries
DATAD: Database of African Theses and Dissertations
LMIC: Low- and middle-income countries
MMAT: Mixed Methods Appraisal Tool
PLWD: People living with dementia
PRISMA: Preferred Reporting Items for Systematic Reviews and Meta-Analysis
SSA: Sub-Saharan Africa
WHO: World Health Organisation
